# Kisameet Clay Isolated from the Central Coast of British Columbia, Canada, Demonstrates Broad-Spectrum Antimicrobial Activity

**DOI:** 10.1128/mBio.00169-16

**Published:** 2016-03-08

**Authors:** George G. Zhanel, James A. Karlowsky

**Affiliations:** Department of Medical Microbiology and Infectious Diseases, College of Medicine, University of Manitoba, Winnipeg, Canada

## Abstract

Clay minerals are naturally occurring layered phyllosilicates which consist of fine particles and possess antimicrobial activity. In a recent article, Behroozian et al. obtained Kisameet clay (KC) from Kisameet, from the central coast of British Columbia, Canada, northwest of Vancouver and assessed its antimicrobial activity versus 16 selected ESKAPE pathogens (*Enterococcus faecium*, *Staphylococcus aureus*, *Klebsiella pneumoniae*, *Acinetobacter baumannii*, *Pseudomonas aeruginosa*, and *Enterobacter* spp.) possessing a variety of different resistance profiles [S. Behroozian, S. L. Svensson, and J. Davies, mBio 7(1):e01842-15, 2016, http://dx.doi.org/10.1128/mBio.01842-15]. KC demonstrated complete bacterial eradication of *Klebsiella pneumoniae*, *Acinetobacter baumannii*, *Pseudomonas aeruginosa*, and *Staphylococcus aureus* within 24 h. For *Enterobacter* spp., the organisms were eradicated with 1% KC within 5 h, while for *Enterococcus faecium*, it took 48 h to kill all organisms. Although many questions need to be answered, these exciting findings highlight the importance of testing natural substances/products from around the globe to assess whether they possess antimicrobial activity and potential for usage as topical, oral, or systemic agents for the treatment of multidrug-resistant pathogens.

## COMMENTARY

Infections caused by antimicrobial-resistant, multidrug-resistant (MDR), and at times extensively drug-resistant (XDR) pathogens are a North American and worldwide crisis. Although many pathogens are resistant to antimicrobials, the ESKAPE group of bacterial pathogens, which includes *Enterococcus faecium*, *Staphylococcus aureus*, *Klebsiella pneumoniae*, *Acinetobacter baumannii*, *Pseudomonas aeruginosa*, and *Enterobacter* spp., are responsible for a large part of health care-acquired infections and are frequently resistant to antimicrobial therapy (1–5).

Researchers have suggested several potential solutions to the problem of antimicrobial-resistant infections, but the discovery and development of new, ideally novel, antimicrobial agents continue to be a critical strategy (6). Although a few new agents effective against multidrug-resistant Gram-negative bacilli have recently been licensed, more antimicrobials and especially agents with novel mechanisms of action are urgently needed (7–10).

Clay minerals are naturally occurring layered phyllosilicates which consist of very fine particles (<2 µm) and possess antimicrobial activity. In a recent article in this journal, Behroozian et al. obtained clay from Kisameet, known as Kisameet clay (KC). KC was obtained from the central coast of British Columbia, Canada, northwest of Vancouver (11). Kisameet clay has been used in this region by local First Nations for centuries for a variety of therapeutic purposes. Kisameet clay displays different properties from other clays by possessing a low mineral content (~24% by weight) and by being dominated by the presence of illite. Most importantly, KC has a significant microbial community (1,000 to 3,000 taxa) that includes *Actinobacteria*, which are known to produce a variety of small molecules that may contribute to the antimicrobial activity of KC.

Behroozian et al. prepared a 1% (wt/vol) KC suspension (10 mg of vacuum-desiccated, ground, autoclaved clay in 1 ml of deionized distilled water) and compared it to controls employing water only (11). The investigators tested the activity of the 1% KC solution against 16 selected ESKAPE pathogens possessing a variety of different resistance profiles, including MDR strains. One percent KC suspensions were inoculated with bacteria to a concentration of 1 × 10^7^ CFU/ml and assessed for viable counts at 0, 5, and 24 h of incubation. One percent KC demonstrated complete bacterial eradication of *K. pneumoniae*, *A. baumannii*, *P. aeruginosa*, and *S. aureus* within 24 h. For *Enterobacter* spp., the organisms were eradicated with 1% KC within 5 h, while for *E. faecium*, it took 48 h to kill all bacteria. These rapid eradication times were in contrast to controls of Gram-negative bacilli displaying a ≤1-log reduction over 24 h, and a 1- to 3-log reduction for *E. faecium* and *S. aureus*.

Many unanswered questions remain about KC, including, what small molecules are present within KC that lead to its broad-spectrum antimicrobial activity. In addition, it will be important to establish whether the antimicrobial activity of KC is concentration dependent. As well, once the active compounds are purified from KC, their mechanisms of action will need to be identified. Presently, no data are available regarding the *in vivo* toxicity of KC whether administered topically, orally, or intravenously. Work will need to be performed to assess whether resistance with any organism develops over time with exposure to KC. In addition, it is important to determine whether KC can enhance the activity or provide synergy with known antimicrobial classes. If KC is able to be administered orally or intravenously to animals, its pharmacokinetics will need to be determined.

Although the findings presented by Behroozian et al. demonstrating broad-spectrum antimicrobial activity of Kisameet clay are preliminary, they do, however, bring significant excitement and encouragement to the research community. It is important for researchers to continue to search for natural substances/products around the globe to assess whether they have antimicrobial activity and potential for usage as topical, oral, or systemic agents for the treatment of multidrug-resistant organisms.
